# Optimal Length of Follow-up for the Detection of Unsuccessful Pediatric Pyeloplasty: A Single-Center Experience

**DOI:** 10.3389/fped.2017.00126

**Published:** 2017-06-01

**Authors:** Utsav K. Bansal, Pankaj P. Dangle, Heidi Stephany, Asad Durrani, Glenn Cannon, Francis X. Schneck, Michael C. Ost

**Affiliations:** ^1^Department of Urology, Children’s Hospital of Pittsburgh, University of Pittsburgh Medical Center, Pittsburgh, PA, United States

**Keywords:** pyeloplasty, follow-up, length, recurrence, ureteropelvic junction obstruction

## Abstract

**Objectives:**

To assess the optimal length of follow-up for patients undergoing both open and minimally invasive pyeloplasties to ensure prompt detection of a recurrent obstruction. There are no standard guidelines on ideal follow-up and imaging post-pediatric pyeloplasty currently.

**Methods:**

A retrospective chart review identified 264 patients (<18 years old) who underwent pyeloplasty for ureteropelvic junction obstruction between April 2002 and December 2014. Ultrasound was obtained every 3–4 months for the first year following pyeloplasty and thereafter at discretion of treating physician. Patient characteristics including symptoms and imaging were reviewed.

**Results:**

Of the 264 patients, 72% were male with mean age of 51 months and follow-up of 26.8 months. Approximately 73% followed up to 3 years. Fourteen patients (5.3%) had a recurrent obstruction. Among the failures, 85% were diagnosed and underwent successful redo pyeloplasty within 3 years. Six infants had a recurrence (43% of all unsuccessful surgeries) and were diagnosed within 3 years of the initial surgery. Patients undergoing a minimally invasive procedure were less likely to be followed for more than 3 years compared to an open procedure (*p* < 0.001). Patients with severe hydronephrosis preoperatively were followed longer (*p* = 0.031). Age at surgery and type of surgical approach (*p* < 0.01) were significant predictors of length of follow-up in a negative binomial regression.

**Conclusion:**

Based on the results, a minimum of 3 years of follow-up is necessary to detect the majority of recurrent obstructions. Those patients who have higher than average lengths of follow-up tend to be younger and/or underwent an open surgical approach.

## Introduction

Pyeloplasty has been the gold standard treatment for ureteropelvic junction (UPJ) obstruction. The widespread use of antenatal ultrasonography has led to an increased detection of hydronephrosis (HN), requiring follow-up by pediatric urologists (1). Approximately one-third of HN cases are due to UPJ obstruction, which if untreated can lead to significant and permanent renal damage (2). Although the success rate of both open and minimally invasive pyeloplasties is >92%, little is known about the optimal length of follow-up following the surgery (3).

There is substantial variation in imaging modality and frequency of follow-up. The lack of standard guidelines for postsurgical follow-up has led to much heterogeneity in length of follow-up and types of imaging among pediatric urologists. Based on the national MarketScan^®^ database, 29% of patients following pyeloplasty did not undergo follow-up imaging 1 year postoperatively. With increased use of minimally invasive procedures, there is decreased postoperative interval screening, though the reason is unclear (4).

Early recognition of failed pyeloplasty has potential to salvage the renal function. Based on a retrospective study, the majority of patients with a failed pyeloplasty were detected and treated within 12 months of initial operation (5).

The purpose of this study was to determine if 12 months of follow-up is adequate to detect the majority of failed pyeloplasties and to evaluate an acceptable length of postoperative follow-up for patients undergoing both open and minimally invasive pyeloplasties.

## Materials and Methods

With help of Center for Assistance in Research Using the eRecord system, patients with International classification of Diseases code (753.21 Congenital obstruction of UPJ) and Current Procedural Terminology code (50400, 50405, 50544) were identified. After Institutional Review Board approval (PR014100422), a retrospective chart review identified 333 patients who underwent pyeloplasty secondary to an UPJ obstruction.

All patients <18 years old undergoing an open or minimally invasive dismembered pyeloplasty due to an UPJ obstruction from April 2002 to December 2014 were included in the study. Sixty-nine patients were excluded due to lack of postoperative follow-up information.

Preoperative characteristics evaluated included gender, age at time of surgery, degree and laterality of HN, history of urinary tract infection (UTI), and history of preoperative stent placement. Patients with symptoms such as abdominal colic, with emesis in absence of any other obvious gastrointestional issues and radiographic finding of HN during the episode were considered as a clinical indicator for obstruction. Postoperative characteristics included type of surgical approach (open, laparoscopic, or robotic), duration of stent, degree of HN at both first and most recent outpatient visits, history of UTI, need for pyeloplasty revision, time until surgical revision, and length of follow-up. The degree of obstruction and preoperative HN was determined based on both functional mercaptoacetyltriglycine (MAG-3) renal scans and abdominal ultrasounds, respectively. Hydronephrosis was graded according to the Society for Fetal Urology postnatal grading system into none (Grade 0), mild (Grade 1 and 2), moderate (Grade 3), and severe (Grade 4) (6).

At our institution, a renal ultrasound is obtained at 1-month follow-up and then 3–4 months postoperatively followed by repeat imaging at 6 and 12 months, then annually until the patient’s HN has improved or is stable. To avoid excessive radiation, functional imaging with MAG-3 renal scan is selectively utilized for worsening HN or related symptoms to assess failed initial pyeloplasty. Therefore, the criteria for termination of follow-up are based on resolution of HN seen mainly on serial ultrasounds beginning at 3 months postoperatively.

### Statistical Analysis

Univariate associations between multiple characteristics were determined using both Chi-squared and Fisher’s exact test. An adjusted negative binomial regression was conducted with duration of follow-up as the primary outcome to identity risk factors for follow-up. A similar subgroup analysis was performed on infants (≤12 months) and patients requiring revision surgery. Statistical analysis was performed using Stata^®^ 14.0 with a *p*-value <0.05 considered significant.

## Results

A total of 264 patients were included in the study. Patient demographics are given in Table 1. The median age was 21 months (1.2–205.6 months). A preoperative stent was required in 14 children (5%) due to worsening HN, infection, or symptomatic presentation.

**Table 1:** **Preoperative and postoperative demographics**.

	No. of patients (%)
**Preoperative characteristics**	
Male	189 (72)
Female	75 (28)
Age (months)	
0–12	102 (39)
12–36	46 (17)
>36	116 (44)
Hydronephrosis (HN)	
Mild	10 (4)
Moderate	44 (17)
Severe	210 (79)
History of preoperative urinary tract infection (UTI)	31 (12)
**Postoperative characteristics**	
Type of surgery	
Open	82 (31)
Laparoscopic	110 (42)
Robotic	72 (27)
Intraoperative stent placed	233 (88)
History of postoperative UTI	17 (6)
Postoperative HN at 3–4 months	
Resolved	59 (22)
Mild	114 (43)
Moderate	71 (27)
Severe	20 (8)
Most recent HN	
Resolved	84 (32)
Mild	132 (50)
Moderate	34 (13)
Severe	14 (5)
Redo pyeloplasty required	14 (5)

Postoperative characteristics are listed in Table 1. Of the 264 patients, 233 (88%) had a stent postoperatively. The median length of postoperative stent duration was 30 days. Those that required a longer duration was due to persistent HN. None of the patients with a longer stent duration required a revision.

In our cohort, 210 patients (79%) had severe HN preoperatively. The majority of the patients (69%) underwent a minimally invasive approach (42% laparoscopic and 27% robotic-assisted). Of the 82 patients (31%) who underwent an open approach, 73 (89%) had severe HN. These patients were more likely to be younger (*p* < 0.001) and have severe HN preoperatively (*p* = 0.008). Only 8% of all patients had severe HN on 3–4 months postoperative imaging as shown in Table 2. Table 2 shows the rates of resolution of HN based on the degree of HN.

**Table 2:** **Rate of resolution of hydronephrosis (HN) based on postoperative renal ultrasound at 3–4 months**.

	Postoperative HN				
Preoperative HN	None	Mild	Moderate	Severe	Total
Mild	4 (40)	6 (60)	0 (0)	0 (0)	10 (100)
Moderate	8 (18)	26 (59)	9 (20)	1 (2)	44 (100)
Severe	47 (22)	82 (39)	62 (29)	19 (9)	210 (100)
Total	59 (22)	114 (43)	71 (27)	20 (8)	264 (100)

Follow-up characteristics are summarized in Table 3. The median duration of follow-up was 16.69 months (3–120 months). The majority of patients (193 patients, 73%) were followed up to 3 years with serial ultrasounds. An additional 42 patients (16%) were followed for up to 5 years with resolution. All the patients who followed up for more than 5 years (29 patients, 11%) had severe HN preoperatively and had either resolution, mild, or moderate HN 3–4 months postoperatively (9, 14, or 6 patients, respectively). Only two patients required a redo pyeloplasty while the other 27 patients were followed with serial ultrasounds till resolution. Patients undergoing a minimally invasive procedure were less likely to be followed longer than 3 years compared to those having an open procedure (22 vs 38 months respectively, *p* < 0.001). The degree of preoperative HN was a significant variable for length of follow-up with those having more severe HN followed for approximately 7 months longer with each increase in severity (*p* = 0.022). Children older than 9 years of age were followed for a shorter duration (17.2 vs 28.9 months, *p* = 0.049).

**Table 3:** **Length of follow-up following pyeloplasty**.

	Mean follow-up (months ± SE)	*p* Value
**Preoperative characteristics**		
Gender		0.54
Male	26.3 ± 1.8	
Female	28.4 ± 2.9	
Age (months)		0.003
0–12	32.2 ± 2.9	
12–36	26.7 ± 3.6	
>36	22.2 ± 1.9	
Hydronephrosis (HN)		0.02
Mild	18.9 ± 4.2	
Moderate	19.7 ± 2.4	
Severe	28.7 ± 18	
History of preoperative urinary tract infection (UTI)	24.6 ± 3.7	0.60
**Postoperative characteristics**		
Overall	26.85 ± 1.54	
Type of surgery		<0.001
Open	37.9 ± 3.6	
Laparoscopic	27.2 ± 2.1	
Robotic	13.7 ± 1.0	
Intraoperative stent placed	25.8 ± 1.6	0.06
History of postoperative UTI	36.3 ± 7.6	0.11
Redo pyeloplasty required	41.5 ± 7.4	0.02

**Figure 1:**
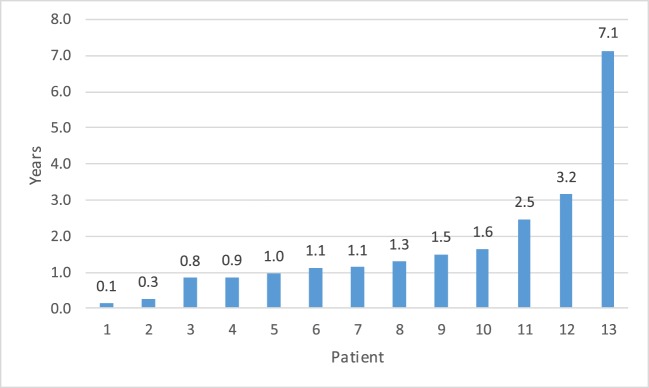
**Time to detection for failed pyeloplasty after initial repair**.^a^ The vast majority of failures (85%) were detected within 3 years. ^a^One patient data missing for time to detection as patient presented to our institution for recurrent obstruction. Initial date of surgery is unknown.

**Figure 2:**
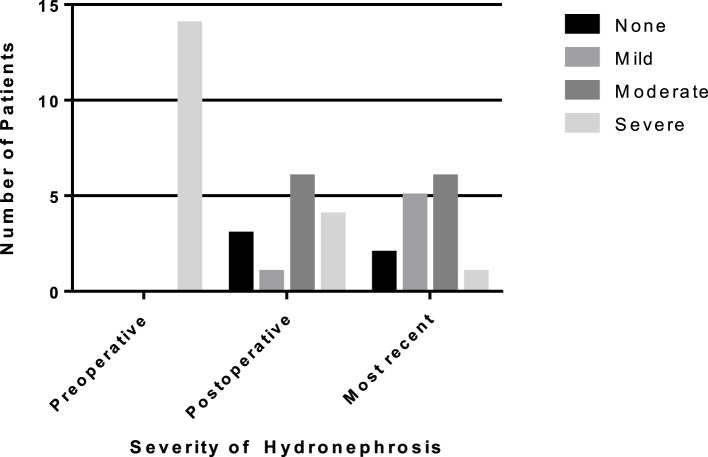
**Rates of resolution of hydronephrosis (HN) from preoperative to most recent ultrasound in patients requiring a redo pyeloplasty**. The number of patients with severe HN decreased to only one patient on most recent ultrasound.

Fourteen patients (5.3%) had a recurrent UPJ obstruction of which one patient presented to our institution initially for a revision surgery. Preoperative and postoperative characteristics are given in Table 4. Of these, 85% were diagnosed and underwent a successful redo pyeloplasty within 3 years while only five failures (38%) were diagnosed with 12 months (Figure 1). The majority (64%) presented with complaints such as abdominal pain, flank pain, or other gastrointestinal symptoms while 36% remained asymptomatic and continued or worsening HN was detected on routine ultrasound. Those that had their redo pyeloplasty done more than 3 years after surgery all presented to clinic with gradual flank pain with nausea and vomiting. A subanalysis revealed that 77% of these failed pyeloplasties were diagnosed and treated within 2 years following the initial surgery and all of these patients had severe HN preoperatively. Only four patients had persistently severe HN 3–4 months postoperatively, while two patients’ HN resolved during the same timeframe (Figure 2). Those requiring a redo pyeloplasty were more likely to be followed longer (41.5 vs. 26.0 months, *p* = 0.024). The intraoperative findings during the redo pyeloplasty revealed a high insertion in 3 patients (23%) and a stricture at the previous anastomotic site in 11 patients (79%). Although these patients required a revision surgery, only four patients (31%) had persistently severe HN 3–4 months after initial surgery. Three patients (23%) saw a resolution in HN while seven patients (54%) saw at least some improvement. As a result, 10 patients developed worsening HN within 3 years of surgery, requiring a redo pyeloplasty.

**Table 4:** **Preoperative and postoperative demographics for patients requiring redo pyeloplasty**.

	No. of patients (%)
**Preoperative characteristics**	
Male	10 (71)
Female	4 (29)
Age (months)	
0–12	6 (43)
12–36	3 (21)
>36	5 (36)
Hydronephrosis	
Mild	0 (0)
Moderate	0 (0)
Severe	14 (100)
Preoperative stent placed	2 (14)
History of preoperative urinary tract infection (UTI)	1 (7)
**Postoperative characteristics**	
Type of surgery	
Open	5 (36)
Mean follow-up (years) ± SE	5.75 ± 1.49
Laparoscopic	8 (57)
Mean follow-up (years) ± SE	2.95 ± 0.44
Robotic	1 (7)
Follow-up (years)	0.76
Intraoperative stent placed	13 (93)
History of postoperative UTI	4 (29)

A negative binominal regression analysis was conducted on preoperative characteristics and concluded that only age at time of surgery (*p* < 0.01) and type of surgical approach (*p* < 0.01) are significant predictors of duration of follow-up. Gender, preoperative HN, and history of preoperative UTI were not found to be significant indicators for length of follow-up.

A secondary subgroup analysis was conducted on patients ≤12 months old. The majority (60%) underwent open pyeloplasty and those with severe HN were more likely to undergo open pyeloplasty (*p* = 0.04). The type of surgical approach continued to be a significant indicator of duration of follow-up (*p* = 0.015). Of the 102 infants, 68% were followed for up to 3 years with a mean time of 32.2 months (3–120 months). Of the 14 patients who required a surgical revision, 6 (43%) were infants with 83% of these recurrences detected and revised within 2 years of initial surgery and 100% by 3 years of surgery.

## Discussion

The ideal duration of follow-up following pediatric pyeloplasty has largely remained unknown. There are no standard guidelines, thus studies have sought to evaluate an acceptable length of follow-up based on single-center experiences. Without existing guidelines, the duration of follow-up is variable and based upon individual physician preferences.

Psooy et al. concluded the ideal follow-up post-dismembered pyeloplasty in 123 pediatric patients was 2 years with an unobstructed diuretic renogram performed 3 months postoperatively with serial ultrasounds as needed (7). van den Hoek et al. concluded that a renal scan showing stable or improved function and enhanced drainage postoperatively at 9 months was evidence of success, and these results were consistent after 5–7 years of follow-up. Prolonged follow-up with repeat renal scan was not justified (8).

In a larger series, Helmy et al. reviewed 590 pyeloplasties of which 18 (3%) had a recurrent UPJ obstruction at a mean period of 26 months (7–48 months) from the initial surgery. Pain was the most common presenting symptom (78%), though all patients had worsening HN and an obstructed pattern on renal scan (9).

We found that the type of surgical approach and age were the most significant predictors determining length of follow-up. The rate of open pyeloplasties has decreased significantly with the advent of a minimally invasive approach (10). Over the past 12 years, our institution has seen a decrease in the number of open procedures with an increase in robotic-assisted laparoscopic pyeloplasty (RALP). In addition, our study provides further evidence that those undergoing minimally invasive pyeloplasties are followed for less time compared to open procedures with equivalent outcomes. Of all patients, 97% of those undergoing robotic procedures were followed for less than 3 years. There are multiple studies examining the efficacy of open versus laparoscopic surgery as well as pure laparoscopic approaches to robotic-assisted (11, 12). Song et al. observed that pediatric patients who undergo a RALP have a decrease in both length of hospital stay and use of pain medication. In addition, there were no significant differences in success rates between the three surgical approaches (13). Barbosa et al. found that in addition to shorter hospital stays, those children who underwent a robotic approach had decreased time to resolution of HN and decreased rates of infection. The authors attributed this decrease in resolution time to more precise movements and less surgical trauma when performing a RALP (14).

Our results also indicated an association between degree of preoperative HN and type of surgical approach, with an increased likelihood of those with severe HN undergoing an open approach. Clinically, however, the type of procedure was less likely to be influenced by severity of HN and more so surgeon preference. In addition, younger patients were more likely to undergo open surgery. This was likely due to the fact that a minimally invasive pyeloplasty is more technically challenging in younger patients.

In addition to the type of surgical approach, age was also a significant indicator of length of follow-up. Younger patients were more likely to be followed for longer than 3 years, regardless of type of surgical approach when age is a continuous variable. Hsi et al. also found younger children to be seen more frequently by health care providers, which could contribute to increased follow-up (4). In addition, those with severe HN are treated at a younger age and may need increased follow-up.

A secondary outcome for evaluating ideal follow-up time was success of pyeloplasty. Almost 80% of all failures were diagnosed and treated within 3 years while only 33% were treated within the first year. As a result, while twelve months of follow-up is not adequate to detect the majority of failed pyeloplasties, 3 years of follow-up is sufficient. The few number of patient that required longer follow-up were more likely to be outliers. The majority of patients with a failed pyeloplasty after 3 years presented with complaints of abdominal and/or flank pain and associated nausea/vomiting. Thus, both children and parents should be counseled to return to clinic if any symptoms develop (5).

In addition to length of follow-up, the type of and interval between imaging has also varied greatly. While some clinicians prefer a functional scan within 1 year of surgery, some rely on serial ultrasounds to detect any recurrence. In a study evaluating the national trends in follow-up imaging, 926 patients were included and approximately one-third of these patients had no additional imaging after 1 year. Of those that did, the vast majority underwent renal ultrasounds only with functional imaging (either renography or excretory urography) being conducted rarely between 0 and 6 months postoperatively (4). In addition, when functional imaging was used, it did not have a significant impact on the likelihood of imaging being done 1 year postoperatively (4).

Similar to the national data, our institution primarily used renal ultrasounds as the imaging modality 3–4 months postoperatively and then every 6–12 months upon the surgeon’s discretion. We were able to follow progression to resolution effectively and diagnose the majority of those requiring a second surgery due to recurring obstruction within three years. Given current practices, there have been minimal guidelines as for appropriate postoperative imaging and for how long patients should be followed. Though a postoperative MAG-3 renal scan would provide more information as to the resolution of HN, the increased healthcare cost and excess exposure to radiation in a setting of high success rate made our institution, among many others, focus primarily on ultrasound imaging. Psooy et al. found that in children who had a non-obstructive renogram 1 year postoperatively, the likelihood of a secondary obstruction after 5 years of follow-up was low. The authors recommended follow-up for 2 years with baseline ultrasounds (7).

There are multiple limitations to our study. This is a single-center, retrospective study lending to inherent selection biases. Multiple surgeon and different surgical approaches is a potential bias, though the goal was to assess the minimum follow required to detect failures. We also do not routinely perform functional imaging post-pyeloplasty. As a result, it is possible that the diagnosis of some of the recurrent cases may have been delayed. We use renal ultrasound in detecting worsening HN as it is our primary modality of evaluating success and avoids additional radiation. We did not assess the frequency of follow-up within the 3-year period following the initial pyeloplasty and, as a result, cannot propose an ideal frequency of follow-up.

Based on our single-center experience, the majority of failed pediatric pyeloplasties are diagnosed within 3 years of the initial repair. Younger patients with severe HN and those undergoing open surgical repair tend to be followed for a longer period. The majority of patients with recurrent obstruction have severe preoperative HN. Guidelines including both the ideal postoperative imaging and length are follow-up are necessary, thus necessitating the need for a prospective, randomized controlled multicenter study to address these critical questions.

## Author Contributions

PD: concept, design, analysis, manuscript writing critical review, and final approval. UB: data collecting, analysis, and manuscript writing. AD: data collection. HS, GC, and FS: patient contribution. MO: critical review.

## Conflict of Interest Statement

None of the contributing authors have any conflict of interest, including specific financial interests or relationships and affiliations relevant to the subject matter or materials discussed in the manuscript.
